# Dental Maturity in Saudi Children Using the Demirjian Method: A Comparative Study and New Prediction Models

**DOI:** 10.1155/2013/390314

**Published:** 2013-02-26

**Authors:** Ziad D. Baghdadi

**Affiliations:** Department of Preventive Dentistry, Riyadh Colleges of Dentistry and Pharmacy, P.O. Box 67126, Riyadh 11596, Saudi Arabia

## Abstract

A sample of 422 dental panoramic radiographs from individuals of known age (from 4 to 14 yrs), sex (males: 217, females: 205), and ethnicity (Saudi) was collected. A dental maturation score for each individual was calculated using the Demirjian method. Age was then estimated using the original Demirjian curves and tables based on French-Canadian population and population-specific curves and tables for Arab (Saudi and Kuwaiti) and European (Belgian) populations. The differences between dental age and chronological age were analyzed and compared using paired *t*-tests, one-way ANOVA test, and a *post hoc* Scheffé's test. The Demirjian method utilizing French-Canadian standards presented significant difference between dental age and chronological age for the total sample and in the vast majority of age groups in both sexes. The mean overestimation of age was about 10 months (*P* < 0.05). The tables designed specifically for Arab populations had a significantly lower error than the tables designed for French-Canadian and Belgian populations. The latter had the largest error in age predication. New age prediction models and maturation scores for Saudi population were developed based on the Demirjian method using multinomial functions.

## 1. Introduction

Several forms of biological age, such as skeletal, morphological, and dental, assess the physiological maturity of a child [1]. Dental age (DA) is an important factor to consider when treating malocclusion or inappropriate growth of the face [1–3]. DA as a means for determining chronological age is valuable in cases of adopted children, children who have committed legal offences, or in forensic cases. A scoring system, such as the Demirjian method, scores the different stages of tooth development resulting in a dental maturity score [4]. 

Systems based on the eruption of teeth are inaccurate methods of determining dental age because eruption is heavily influenced by environmental factors [3]. Tooth development is less affected by dental arch space, extraction of deciduous teeth, or tipping or impaction of teeth, which may influence the eruption process [3]. Reliable events in dental development, such as tooth calcification, allow for improved prediction of dental maturity [2]. The Demirjian method is highly accurate when evaluating young children (<6.5), less so with older children [5]. 

In a study [6] that compared dental age to chronological age in Somali children to that of matched white Caucasian children in England, the mean difference found between dental age and chronological age was 1.01 years for Somali boys, 0.19 for Caucasian boys, 1.22 years for Somali girls, and 0.52 years for Caucasian girls. Somali children appear to be significantly more dentally mature than their Caucasian peers. Similarly, another study [7] tested the accuracy of the dental age estimation methods of Moorrees et al. and Demirjian on children of different ethnic groups in South Africa. Because the study found that the Moorrees et al. method consistently underestimated age and the Demirjian method overestimated age, dental age tables were developed specifically for these ethnic groups. When tested, these tables were found to be more accurate than either the Moorrees et al. or the Demirjian methods [8]. These findings suggest a need for population-specific dental development standards based on ethnicity to improve the accuracy of dental age assessment. The purpose of this cross-sectional study was to develop age prediction models for children, using the original Demirjian scores, by testing accuracy of the scores in Saudi Arabian children by comparing the dental age of different population-specific curves to the children' chronological ages. 

## 2. Methods

The sample, 422 panoramic radiographs of 217 boys and 205 girls, was collected from university-based and private-based pediatric dental clinics. Children with systemic diseases that can affect development of teeth, mandibular hypodontia (except third molars), and low-quality radiographs were excluded. The children's ages ranged from 4 to 14 years. 

The radiographs were divided into 10 groups by the child's chronological age, calculated by subtracting the date of the radiograph from the date of birth. Each group was comprised of radiographs from children of the same age (children were grouped by a span of 1 year starting from 4 years up to 14). Dental age assessment was performed according to the Demirjian method [1]. Briefly, the development of each left permanent mandibular tooth, except the third molar, was rated on an 8-stage scale from A to H, and the criteria for the stages were given separately for each tooth. Each stage of the seven teeth was scored, and the sum of the scores resulted in an evaluation of the child's dental maturity, measured on a scale from 0 to 100. The score of each child was then converted to dental age using standard tables for both boys and girls.

The data were stored and analyzed using statistical software SPSS ver. 19 (IBM Corp., Somers, NY, USA) and Minitab ver. 16 (Minitab Inc., State College, PA, USA). Paired *t*-tests were used to establish any differences between estimated ages obtained by the Demirjian scores and chronological ages for both sexes. The difference between dental age and chronological age using the different age groups for each gender was tabulated according to previously published tables calculated using a modified Demirjian method for European (Belgian) [9], other Arab (Kuwaiti) [10], and Saudi [11] children. Differences were compared using a one-way ANOVA and a *post hoc* Scheffé's test. Regression models explored calculations of age (taken as the dependent or response variable) and maturity score or dental age (taken as the independent or explanatory variables). Calculations were done separately for boys and girls. Level of significance was set at *P* = 0.05. 

Prior to collecting age assessment data, a reliability study assessed the magnitude of the intraobserver errors of interpretation and detection. Two calibrated examiners assessed the maturation stage of the seven left mandibular permanent teeth without the knowledge of chronological age or gender. To evaluate reproducibility, 25 radiographs (with 175 tooth-individual ratings) were randomly selected and assessed by both examiners. There were approximately four weeks between the two rating sessions. Later, the author was the only rater for the developmental stages of the teeth. 

## 3. Results

The Cronbach's alpha between the first rating and the second rating was 0.994, indicating a high level of reproducibility. 

Using the Demirjian method, DA, CA, and differences between DA and CA (DA-CA) for both genders and all age groups are presented in Table 1. The paired *t*-test results indicated that the mean CA was 8.89 and the mean DA was 9.69. This mean indicated an overaging of the sample as by about 10 months, which held equally true for both sexes. The mean age difference was 0.77 (SD 0.85, CI 0.65–0.88) in boys and 0.83 (SD 0.79, CI 0.72–0.94) in girls. The mean differences between DA and CA were extremely statistically significant (*P* < 0.001), and therefore corrections for multiple comparisons were not used. The mean difference between the DA and CA ranged from −0.12 to 1.21 yrs in boys and from 0.42 to 1.26 yrs in girls. The differences in the means were statistically significant for all age groups and genders, except in 8-year-old, 11-year-old, and 13-year-old boys. 

**Figure 1:**
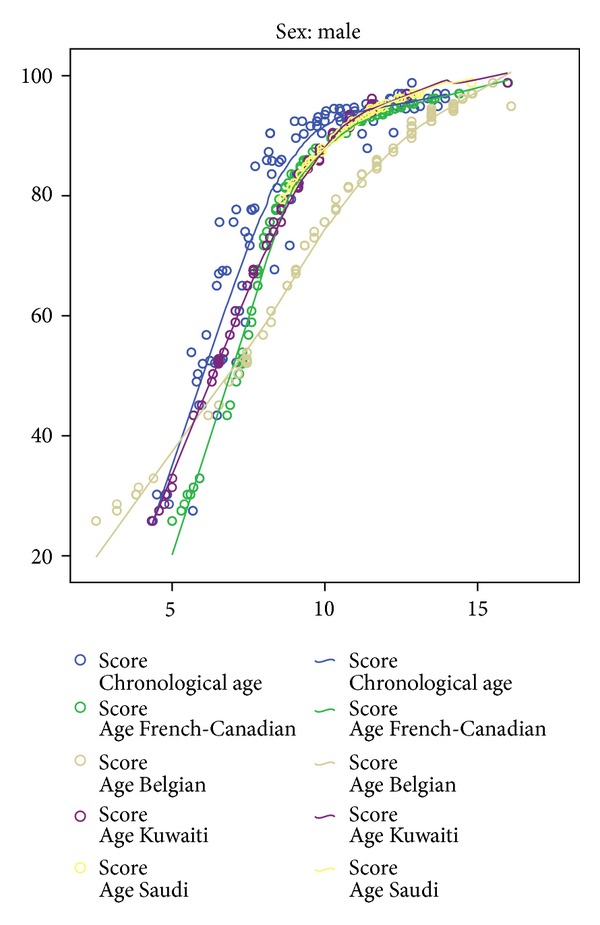
Comparison of the study population to the different existing curves—boys.

**Figure 2:**
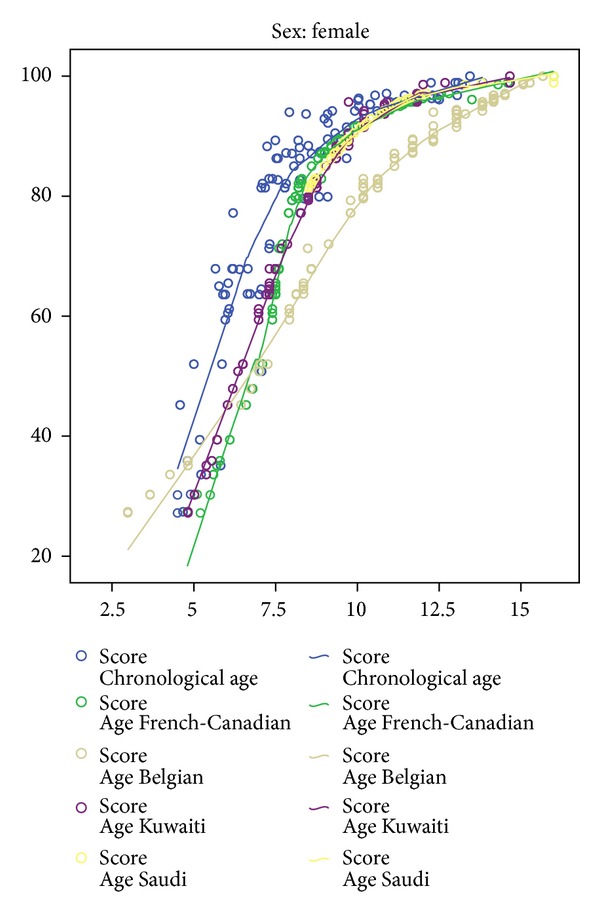
Comparison of the study population to the different existing curves—girls.

Tables 2 and 3 present the differences between the DA-CA when assessed using population-specific curves, taking age group as a factor. From the results, it appears that the original Demirjian method and its modifications consistently overestimated the age of the sample. One-way ANOVA found significant differences in estimation between the different methods for both boys (*F* = 127.88, *P* < 0.001) and girls (*F* = 136.58, *P* < 0.001). However, *post hoc* tests revealed that the mean difference in estimation based on the Kuwaiti and Saudi curves for boys was not statistically significant (Table 4). For girls, the mean differences between the Kuwaiti, Saudi, and the original French-Canadian curves were not statistically significant (Table 5). Scheffé's homogeneous subsets found that when boys were compared, the tables designed specifically for Arab (Kuwaiti and Saudi) populations had a significantly lower error than the tables designed for Caucasian + Amerindian (French-Canadian) and European (Belgian) populations (Table 6). A similar comparison between girls found that there were no statistical differences between the original Demirjian method and the curves designed for Arab populations (Table 7). Figures 1 and 2 show the fitting curve for the study population as compared to the three existing curves for boys and girls.

**Figure 3:**
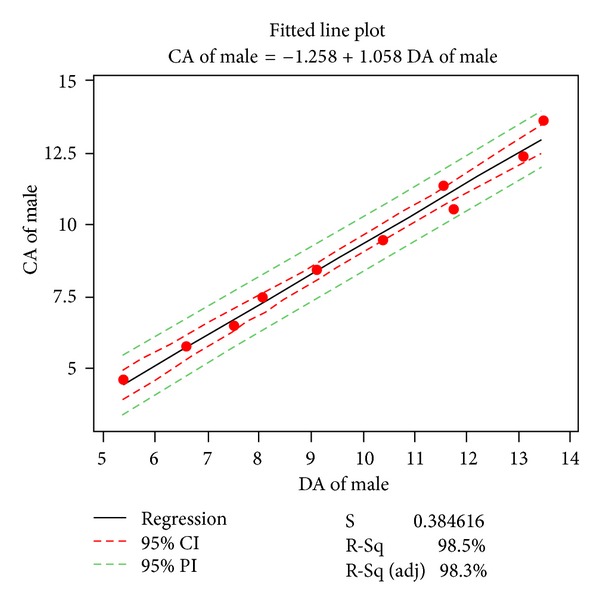
Regression of mean chronological versus estimated ages of our Saudi males with 95% confidence and prediction intervals; *R* ^2^ = 0.985.

**Figure 4:**
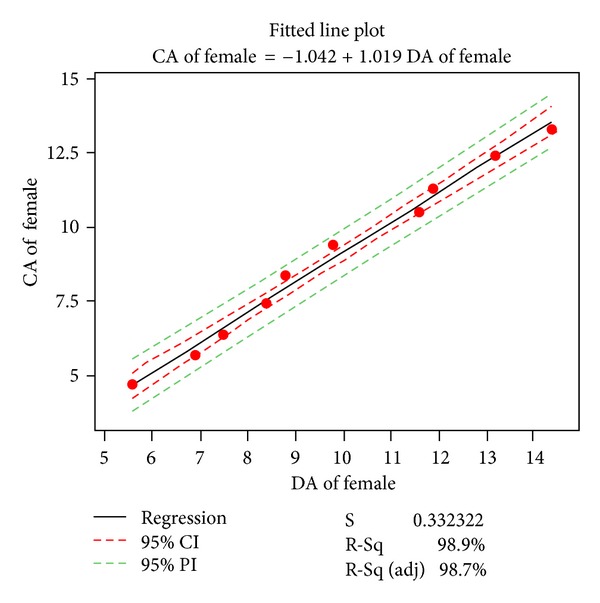
Regression of mean chronological versus estimated ages of our Saudi females with 95% confidence and prediction intervals; *R* ^2^ = 0.989.

**Figure 5:**
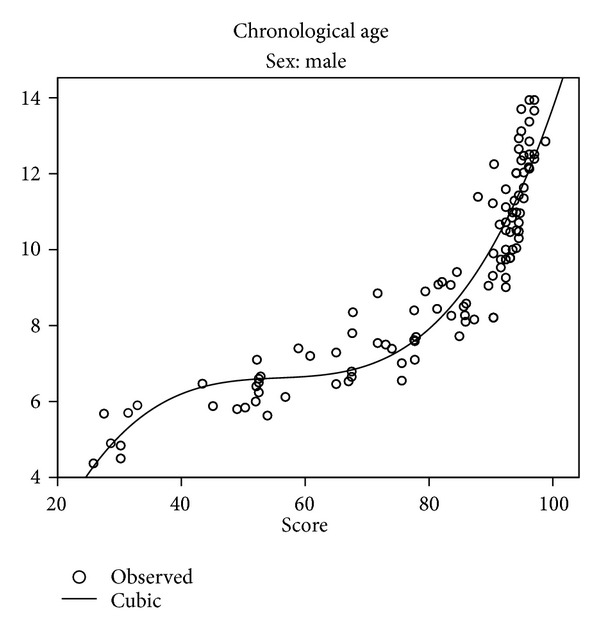
Scatter plots of maturity score against chronological age in Saudi boys.

**Figure 6:**
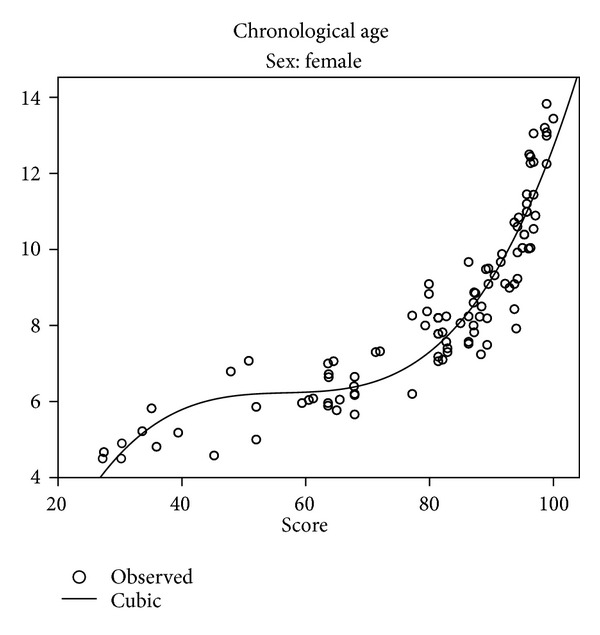
Scatter plots of maturity score against chronological age in Saudi girls.

Missing data for age groups younger than 7.5 years made the comparison between the present sample and existing curves designed for Saudi population impossible. Different relationships between chronological age, on one hand, and dental age were explored. Two models: the linear and the cubic were selected. Figures 3 and 4 present linear regressions for chronological age versus estimated ages of boys and girls with 95% confidence and prediction intervals. The equation can be used to estimate mean age based on the Demirjian dental age.  Table 8 and Figures 5 and 6 present the cubic functions between age and maturation scores. New gender-specific dental maturity tables were developed based on a third-degree regression because it proved to be the best fit to the plots (Tables 9 and 10). 

## 4. Discussion 

Dental and skeletal developments provide measures of physiological age to predict the optimal timing for treatment in orthodontic, orthopedic, or pediatric clinical practice or to estimate the chronological age of skeletal remains in forensic or archeological contexts [12]. Dental development is less affected by environmental quality than skeletal development [12]. 

Several methods have been proposed for assessing dental development, which is generally referred to as dental aging. Dental aging appears in two forms: calcification (tooth development) and eruption patterns [13]. Eruption refers to emergence of the tooth through the gum, rather than to emergence from the bone or reaching the occlusal plane [13]. This makes it impossible to use eruption for age estimation on skeletal remains in forensics. Tooth emergence may be influenced significantly by local exogenous factors, such as infection, obstruction, crowding, and premature extraction of the deciduous predecessor or adjacent permanent teeth [12]. Most of the disadvantages can be avoided by using stages of tooth formation from radiographical data on the calcification of teeth to determine dental maturity from in utero until the late twenties, if the third molar is used. 

The Demirjian eight-stage method is one of the principal methods used to quantify the degree of maturity from the age from 3 to 17 years [1]. Although the Demirjian method performs well in terms of observer agreement and correlation between the estimated age and true age [14] (which is in agreement with the current study), the Demirjian original French-Canadian standards do not accurately estimate the chronological age in all samples [1, 15–19].

It is important to remember that the difference in chronological age and dental age may be attributed to different factors, including the accuracy of the method, examiner's training and experience, sample size and distribution, and statistic approach to the obtained results [20]. However, it is equally important to realize that no age estimation will accurately determine the exact age for every individual as development naturally varies between individuals. Forensic science uses age ranges when estimating age for just this reason [13]. Differences between real age and estimated age up to 12 months were considered to be within normal standards for some authors [21]; however, smaller intervals are strived for by other authors [22], hence the construction of the “population-specific Demirjian curves.”

The results of the current study corroborate the results of previous studies [10, 12] that examined the applicability of the Demirjian method to similar populations. A study [11] assessing the dental age in Saudi children aged from 8.5 to 17 years found that Saudi children from Riyadh were overestimated by 0.3 years for boys and 0.4 years for girls. Similar results were reported on Kuwaiti children aged from 3 to 14 yrs, but the overestimations were 0.71 yrs for boys and 0.67 for girls [10]. The samples constituting the three studies from Saudi Arabia and Kuwait are from the same ancestry, geographically close to each other, and exposed to similar dietary and behavior patterns [23]. The only difference is that the sample in the Al-Emran study was somewhat larger (*N* = 490) and older in age (from 7.5 to 17 yrs). The present study has found that overall overestimations are statistically significant, but that when comparing age groups in the three studies, the results were less consistent as some groups showed significant differences between true age and estimated age, and some did not. 

The results here confirm the necessity of developing specific scores or curves for specific populations, as agreed by most authors [22]. The regression models used here resemble those proposed by Cruz-Landeira et al. [24] and differ from most of the previously published researches [3] considering age as the independent variable and the score as the dependent. Here, we inverted the variables, considering the chronological age (parameter that is wanted to be calculated in a real forensic case) as a function of the maturity score (the known parameter). The cubic model proved to be the best as, after exploring several models, it provided the best fit between maturation scores and chronological age (Figures 5 and 6). 

Although the sample size of the current study seems smaller than that in similar studies, this is not necessarily a limitation in forensic cases [25]. Power analysis for each age group in our study ranged from 0.88 to 1.0. In addition, the results of this study corroborate the results of our previous study [26] which had a smaller sample (*N* = 176). Although Scheffé's procedure followed here is the most popular, flexible, and conservative of the *post hoc* procedures, it is also the least statistically powerful procedure because it involves contrasts of more than two means at the same time. However, due to the extreme significant (or nonsignificant) results shown in Tables 4 and 5, it is unlikely that the test leads to Type II errors [27]. 

As presented in Figures 5 and 6, a 100% maturity is achieved at a mean age of 13.2 for males and 12.7 for females. This suggests that the Demirjian method is inadequate after the age of 13 in Saudi population. Other researchers [24] have reported that 100% of maturity was achieved for girls at the age of 12 in a Spanish sample and at the age of 14.1 in a Venezuelan sample; boys showed a median growth delay of 1 year compared to girls. The gender difference is most likely biological and as most maturation events (e.g., height, and sexual maturation) is faster in girls. This is in agreement with this study, where girls were dentally more advanced than boys. 

After an evaluation of findings to the literature, it may be concluded that although over- and underestimations result from the Demirjian method, it remains a valuable way to evaluate the age of a child based on dentition. The Demirjian method may not yield an exact age in every case; however, it seems to be a clinically acceptable method to study the pattern of growth within a certain population (e.g., normal children versus children with disabilities) or between different populations. As new curves for populations are more accurate than the original curves, new curves were developed which require further validation studies. 

## Figures and Tables

**Table 1:** Dental age (DA), chronological age (CA), and difference between dental age and chronological age.

Age group	Gender	Mean (SD)			95% CI	*P* value
		CA (SD)	DA (SD)	DA − CA (SD)	DA–CA	
4.00–4.99	M (7)	4.62 (0.23)	5.37 (0.26)	0.75 (0.18)	0.58–0.93	0.000
	F (9)	4.66 (0.14)	5.57 (0.68)	0.90 (0.72)	0.34–1.46	0.006
5.00–5.99	M (12)	5.77 (0.10)	6.60 (0.80)	0.83 (0.79)	0.33–1.33	0.004
	F (20)	5.63 (0.34)	6.90 (0.77)	1.26 (0.66)	0.95–1.58	0.000
6.00–6.99	M (26)	6.46 (0.22)	7.50 (0.35)	1.04 (0.35)	0.90–1.18	0.000
	F (22)	6.35 (0.28)	7.49 (0.25)	1.13 (0.45)	0.93–1.33	0.000
7.00–7.99	M (28)	7.43 (0.25)	8.08 (0.46)	0.66 (0.42)	0.5–0.82	0.000
	F (40)	7.41 (0.29)	8.40 (0.83)	0.98 (0.69)	0.76–1.20	0.000
8.00–8.99	M (26)	8.40 (0.24)	9.10 (0.83)	0.70 (0.98)	0.29–1.09	0.058
	F (38)	8.38 (0.30)	8.80 (0.75)	0.42 (0.69)	0.19–0.65	0.001
9.00–9.99	M (30)	9.43 (0.31)	10.40 (0.98)	0.97 (0.85)	0.65–1.28	0.000
	F (24)	9.42 (0.30)	9.78 (0.82)	0.36 (0.83)	0.01–0.71	0.044
10.00–10.99	M (30)	10.54 (0.33)	11.76 (0.51)	1.21 (0.56)	1.00–1.42	0.000
	F (24)	10.53 (0.35)	11.59 (0.61)	1.05 (0.70)	0.75–1.35	0.000
11.00–11.99	M (16)	11.37 (0.17)	11.55 (1.09)	0.17 (1.05)	−0.38–0.73	0.523
	F (6)	11.36 (0.12)	11.90 (0.46)	0.53 (0.42)	0.09–0.97	0.026
12.00–12.99	M (30)	12.40 (0.31)	13.10 (1.27)	0.69 (1.18)	0.25–1.14	0.003
	F (12)	12.45 (0.26)	13.20 (1.15)	0.74 (1.05)	0.07–1.41	0.033
13.00–13.99	M (12)	13.62 (0.30)	13.50 (0.81)	−0.12 (0.69)	−0.56–0.32	0.557
	F (10)	13.32 (0.30)	14.40 (1.17)	1.08 (1.06)	0.31–1.84	0.011

Total	M (217)	9.27 (2.43)	10.04 (2.44)	0.76 (0.85)	0.65–0.88	0.000
	F (205)	8.48 (2.27)	9.31 (2.30)	0.83 (0.78)	0.72–0.94	0.000

TOTAL	M + F (422)	8.89 (2.38)	9.69 (2.40)	0.80 (0.82)	0.72–0.87	0.000

**Table 2:** Mean difference (±standard deviation) between dental age based on the Demirjian method and chronological age as determined by the different methods in boys.

Age group (*n*)	French-Canadian (SD)	Belgian (SD)	Kuwaiti (SD)	Saudi (SD)
4-5 (7)	0.75 (0.18)	−1.26 (0.53)	0.03 (0.17)	NA
5-6 (12)	0.82 (0.78)	0.11 (1.67)	0.04 (0.79)	NA
6-7 (26)	1.04 (0.35)	1.57 (0.96)	0.51 (0.70)	NA
7-8 (28)	0.66 (0.42)	2.11 (1.01)	0.60 (0.69)	1.68 (0.00)
8-9 (26)	0.69 (0.98)	2.81 (1.33)	0.85 (0.92)	1.22 (0.77)
9-10 (30)	0.97 (0.84)	3.23 (0.82)	0.83 (0.57)	0.95 (0.68)
10-11 (30)	1.21 (0.56)	3.05 (0.48)	0.60 (0.48)	0.80 (0.43)
11-12 (16)	0.17 (1.05)	2.14 (0.65)	−0.39 (0.62)	−0.19 (0.67)
12-13 (30)	0.69 (1.18)	1.91 (0.53)	−0.20 (1.15)	−0.19 (0.88)
13-14 (12)	−0.12 (0.69)	1.20 (0.86)	−1.51 (0.63)	−1.22 (0.41)

Total (217)	0.76 (0.85)	2.12 (1.36)	0.30 (0.96)	0.40 (1.00)

**Table 3:** Mean difference (±standard deviation) between dental age based on the Demirjian method and chronological age as determined by the different methods in girls.

Age group (*n*)	French-Canadian (SD)	Belgian (SD)	Kuwaiti (SD)	Saudi (SD)
4-5 (9)	0.90 (0.72)	−0.36 (1.41)	0.62 (0.52)	NA
5-6 (20)	1.26 (0.66)	1.38 (1.36)	0.95 (0.69)	NA
6-7 (22)	1.13 (0.45)	1.96 (0.85)	0.92 (0.62)	NA
7-8 (40)	0.98 (0.65)	2.82 (1.22)	1.24 (0.82)	1.51 (0.57)
8-9 (38)	0.42 (0.69)	2.73 (0.83)	0.76 (0.53)	0.90 (0.57)
9-10 (24)	0.36 (0.83)	2.95 (1.02)	0.56 (0.67)	0.60 (0.65)
10-11 (24)	1.05 (0.70)	3.28 (0.68)	0.50 (0.90)	0.76 (0.56)
11-12 (6)	0.53 (0.42)	2.68 (0.04)	0.15 (0.34)	0.17 (0.27)
12-13 (12)	0.74 (1.05)	2.03 (0.47)	0.25 (1.35)	−0.09 (0.98)
13-14 (10)	1.08 (1.06)	1.79 (0.41)	−0.15 (1.34)	0.88 (1.43)

Total (205)	0.83 (0.78)	2.40 (1.26)	0.74 (0.85)	0.84 (0.84)

**Table 4:** A *post hoc* test comparing several methods for age estimation in boys.

DiffageMale Scheffe		Multiple comparisons				
(I) group	(J) group	Mean difference (I − J)	Std. error	Sig.	95% confidence interval	
					Lower bound	Upper bound
French-Canadian	Belgian	−1.35373*	.10256	.000	−1.6411	−1.0664
	Kuwaiti	.46452*	.10256	.000	.1772	.7518
	Saudi	.36993*	.11632	.018	.0441	.6958

Belgian	French-Canadian	1.35373*	.10256	.000	1.0664	1.6411
	Kuwaiti	1.81825*	.10256	.000	1.5309	2.1056
	Saudi	1.72366*	.11632	.000	1.3978	2.0495

Kuwaiti	French-Canadian	−.46452*	.10256	.000	−.7518	−.1772
	Belgian	−1.81825*	.10256	.000	−2.1056	−1.5309
	Saudi	−.09459	.11632	.882	−.4205	.2313

Saudi	French-Canadian	−.36993*	.11632	.018	−.6958	−.0441
	Belgian	−1.72366*	.11632	.000	−2.0495	−1.3978
	Kuwaiti	.09459	.11632	.882	−.2313	.4205

*The mean difference is significant at the 0.05 level.

**Table 5:** A *post hoc* test comparing several methods for age estimation in girls.

DiffageFemale Scheffe		Multiple comparisons				
(I) group	(J) group	Mean difference (I − J)	Std. error	Sig.	95% confidence interval	
					Lower bound	Upper bound
French-Canadian	Belgian	−1.57024*	.09554	.000	−1.8379	−1.3026
	Kuwaiti	.09220	.09554	.818	−.1755	.3599
	Saudi	−.00907	.10794	1.000	−.3115	.2934

Belgian	French-Canadian	1.57024*	.09554	.000	1.3026	1.8379
	Kuwaiti	1.66244*	.09554	.000	1.3948	1.9301
	Saudi	1.56118*	.10794	.000	1.2587	1.8636

Kuwaiti	French-Canadian	−.09220	.09554	.818	−.3599	.1755
	Belgian	−1.66244*	.09554	.000	−1.9301	−1.3948
	Saudi	−.10126	.10794	.830	−.4037	.2012

Saudi	French-Canadian	.00907	.10794	1.000	−.2934	.3115
	Belgian	−1.56118*	.10794	.000	−1.8636	−1.2587
	Kuwaiti	.10126	.10794	.830	−.2012	.4037

*The mean difference is significant at the 0.05 level.

**Table 6:** Scheffe's *post hoc* homogeneous subsets showing overall differences between the methods used at *P* = 0.05 for boys.

Method used (*n*)	Subset for alpha = 0.05		
	a*	b*	c*
Kuwaiti (217)	0.30		
Saudi (138)	0.39		
French-Canadian (217)		0.76	
Belgian (217)			2.12

Significance	0.86	1.00	1.00

*Values that are not significantly different based on the *post  hoc* Scheffé contrast will have the same superscript, and values that are significantly different will have different superscripts.

**Table 7:** Scheffe's *post hoc* homogeneous subsets showing overall differences between the methods used at *P* = 0.05 for girls.

Method used (*n*)	Subset for alpha = 0.05	
	a*	b*
Kuwaiti (205)	0.74	
Saudi (132)	0.84	
French-Canadian (205)	0.83	
Belgian (205)		2.40

Significance	0.80	1.00

*Values that are not significantly different based on the *post  hoc* Scheffé contrast will have the same superscript, and values that are significantly different will have different superscripts.

**Table 8:** Cubic equations for boys and girls.

*Y* = −7.424 + 0.741*x* − 0.013*x* ^2^ + 0.00007863*x* ^3^ (males)
*Y* = −8.269 + 0.757*x* − 0.013*x* ^2^ + 0.00007782*x* ^3^ (females)

*Y* is age, and *x* is maturation score.

**Table 9:** Predicted age per maturity score in Saudi boys using the developed function formula based on the Demirjian method.

25.8	4.28746	85.6	8.92035
27.5	4.63997	85.8	8.96403
28.6	4.84737	85.9	8.98608
30.2	5.12152	86	9.00827
31.4	5.30676	87.3	9.30993
32.9	5.51512	87.9	9.45756
43.4	6.38282	89.6	9.90584
45.1	6.4484	90.3	10.10369
49	6.54762	90.4	10.1326
50.3	6.56851	90.5	10.16168
52	6.58939	91.4	10.43079
52.1	6.59044	91.6	10.49243
52.2	6.59147	92.4	10.74578
52.8	6.5973	93.1	10.97653
53.9	6.60675	93.4	11.07805
56.8	6.62817	93.5	11.11225
58.9	6.64555	93.8	11.21591
60.8	6.66675	94.1	11.32118
65	6.74862	94.5	11.46407
67	6.81243	94.7	11.53661
67.5	6.83155	95.3	11.75866
67.6	6.83554	96.1	12.06518
67.7	6.83958	96.2	12.10434
71.7	7.05245	97	12.42458
73	7.14604	98.8	13.19098
74	7.22733		
75.6	7.37547		
77.6	7.59454		
77.7	7.60654		
77.9	7.63085		
79.4	7.8268		
81.3	8.11119		
81.5	8.1436		
82.1	8.24375		
83.5	8.49501		
83.6	8.51392		
84.5	8.69009		
84.9	8.77189		

**Table 10:** Predicted age per maturity score in Saudi girls using the developed function formula based on the Demirjian method.

27.2	4.08377	88.1	8.75631
27.4	4.12524	88.3	8.80327
30.2	4.64983	88.4	8.82697
30.3	4.66671	89.1	8.99705
33.6	5.15701	89.3	9.04699
35.1	5.3399	89.5	9.09754
35.9	5.42803	90.5	9.35954
39.4	5.74337	91.5	9.63728
45.2	6.05669	91.7	9.69475
47.9	6.13495	92.2	9.84131
50.8	6.18559	92.9	10.05345
52	6.19902	93.7	10.30601
59.4	6.24147	94	10.40356
60.5	6.2489	94.2	10.46946
61.2	6.25469	94.4	10.53605
63.6	6.28319	95	10.74007
63.6	6.28319	95.3	10.84448
63.7	6.28473	95.7	10.98622
64.5	6.29826	96	11.09443
65	6.30787	96.1	11.13086
65.5	6.31843	96.3	11.20428
67.8	6.38106	96.8	11.39107
67.9	6.38436	97.1	11.50539
71.3	6.53046	98.6	12.10261
72	6.56966	98.9	12.22728
77.2	6.98061	100	12.69963
79.3	7.21594		
79.6	7.25324		
79.9	7.29151		
81.4	7.49764		
82.1	7.60258		
82.7	7.69714		
82.9	7.72963		
85	8.10086		
86.3	8.35951		
87.1	8.53019		
87.2	8.55216		
87.4	8.59652		
